# Global microRNA expression profile in laryngeal carcinoma unveils new prognostic biomarkers and novel insights into field cancerization

**DOI:** 10.1038/s41598-022-20338-w

**Published:** 2022-10-12

**Authors:** Todor M. Popov, Gergana Stancheva, Silva G. Kyurkchiyan, Veronika Petkova, Stiliana Panova, Radka P. Kaneva, Diana P. Popova

**Affiliations:** 1 Department of ENT, Medical University - Sofia, Adriana Budevska Str 10, 1463 Sofia, Bulgaria; 2 Molecular Medicine Center, Medical University of Sofia, Sofia, Bulgaria

**Keywords:** Cancer microenvironment, Head and neck cancer, Oncogenes, Tumour biomarkers, Tumour-suppressor proteins, Prognostic markers, Biomarkers, Outcomes research

## Abstract

Laryngeal carcinoma is still a worldwide burden that has shown no significant improvement during the last few decades regarding definitive treatment strategies. The lack of suitable biomarkers for personalized treatment protocols and delineating field cancerization prevents further progress in clinical outcomes. In the light of this perspective, MicroRNAs could be promising biomarkers both in terms of diagnostic and prognostic value. The aim of this prospective study is to find strong prognostic microRNA biomarkers for advanced laryngeal carcinoma and molecular signatures of field cancerization. Sixty patients were enrolled and four samples were collected from each patient: tumor surface and depth, peritumor normal mucosa, and control distant laryngeal mucosa. Initially, a global microRNA profile was conducted in twelve patients from the whole cohort and subsequently, we validated a selected group of 12 microRNAs with RT-qPCR. The follow-up period was 24 months (SD ± 13 months). Microarray expression profile revealed 59 dysregulated microRNAs. The validated expression levels of miR-93-5p (χ^2^(2) = 4.68, log-rank *p* = 0.03), miR-144-3p (χ^2^(2) = 4.53, log-rank *p* = 0.03) and miR-210-3p (χ^2^(2) = 4.53, log-rank *p* = 0.03) in tumor samples exhibited strong association with recurrence-free survival as higher expression levels of these genes predict worse outcome. Tumor suppressor genes miR-144-3p (mean rank 1.58 vs 2.14 vs 2.29, *p* = 0.000) and miR-145-5p (mean rank 1.57 vs 2.15 vs 2.28, *p* = 0.000) were significantly dysregulated in peritumor mucosa with a pattern of expression consistent with paired tumor samples thus revealing a signature of field cancerization in laryngeal carcinoma. Additionally, miR-1260b, miR-21-3p, miR-31-3p and miR-31-5p were strongly associated with tumor grade. Our study reports the first global microRNA profile specifically in advanced laryngeal carcinoma that includes survival analysis and investigates the molecular signature of field cancerization. We report two strong biomarkers of field cancerization and three predictors for recurrence in advance stage laryngeal cancer.

## Introduction

Head and neck cancer and laryngeal carcinoma in particular is still a worldwide burden that has shown no significant improvement during the last few decades regarding definitive treatment strategies and earlier diagnosis. Despite recent advances in biological therapy, the survival rate still does not differ from the results achieved decades ago, and curative treatment options remain limited to surgery and/or chemoradiation therapy based on anatomic location and TNM staging^1,2^. The lack of suitable biomarkers in this field ensue failure in defining subgroups eligible for more individualized treatment protocols, something that has already been established in clinical practice for many other solitary malignancies. Field cancerization is another major issue in head and neck cancer that requires considerable effort in terms of research and translational clinical application. The concept behind this theory states that most malignancies arise from contiguous monoclonal preneoplastic fields in the mucosa, and a great number of recurrences originate from remnants of these clonal fields left behind after tumor resection^3^. Since field cancerization can occur without associated morphological change, conventional pathological methods neither can delineate these areas nor access the stochastic risk they carry. Taking this into account, several authors consider the necessity of discovering certain molecular signatures that might be the key to the precise mapping of these fields^4^. In light of these perspectives, our study was designed to investigate the possible polyvalent role of microRNAs both as predictors of survival in tumor tissue and as biomarkers of dysregulation in peritumor laryngeal mucosa, e.g., their possible role as signatures of field cancerization. MicroRNAs inherently could possess such value, since they are small non-coding RNAs with numerous regulatory functions in cancer cell processes. The first reported oncomiR in the literature was described by Calin GA et al. in 2002 in B-cell chronic lymphocytic leukemia cells^5^, and consequently, microRNAs have been a major focus in cancer research due to their strong impact on cascades that are vital for tumor proliferation, invasion and other hallmarks of cancer.

Our study reports the first global microRNA profile specifically in advanced laryngeal carcinoma that includes survival analysis and investigates the molecular signature of field cancerization. The study is a prospective prognostic study in patients with advanced laryngeal carcinoma. All expression levels were compared to paired control samples of healthy distant laryngeal mucosa, which adds statistical power as well as accuracy of fold change^6^. Finally, tumor heterogeneity was addressed by validating all results both in tumor depth and surface.

## Results

The study group included predominantly HPV-negative tumors (90.3%), validated with p16 immunohistochemistry, with all patients having a history of long-term smoking. In terms of tumor staging, the majority of the cases (87.1%) were classified as pT4a, one case was pT4b, and the rest were staged as pT3. Almost half of the group, 48.3% of the cases, had pathologically verified metastatic processes, and the distribution of N status was as follows: N1 (28.6%); N2a/N2b/N2c (57.1%), N3 (14.3%).

### Microarray profiling reveals a consistent microRNA expression pattern between peritumor and laryngeal carcinoma samples

**Figure 1:**
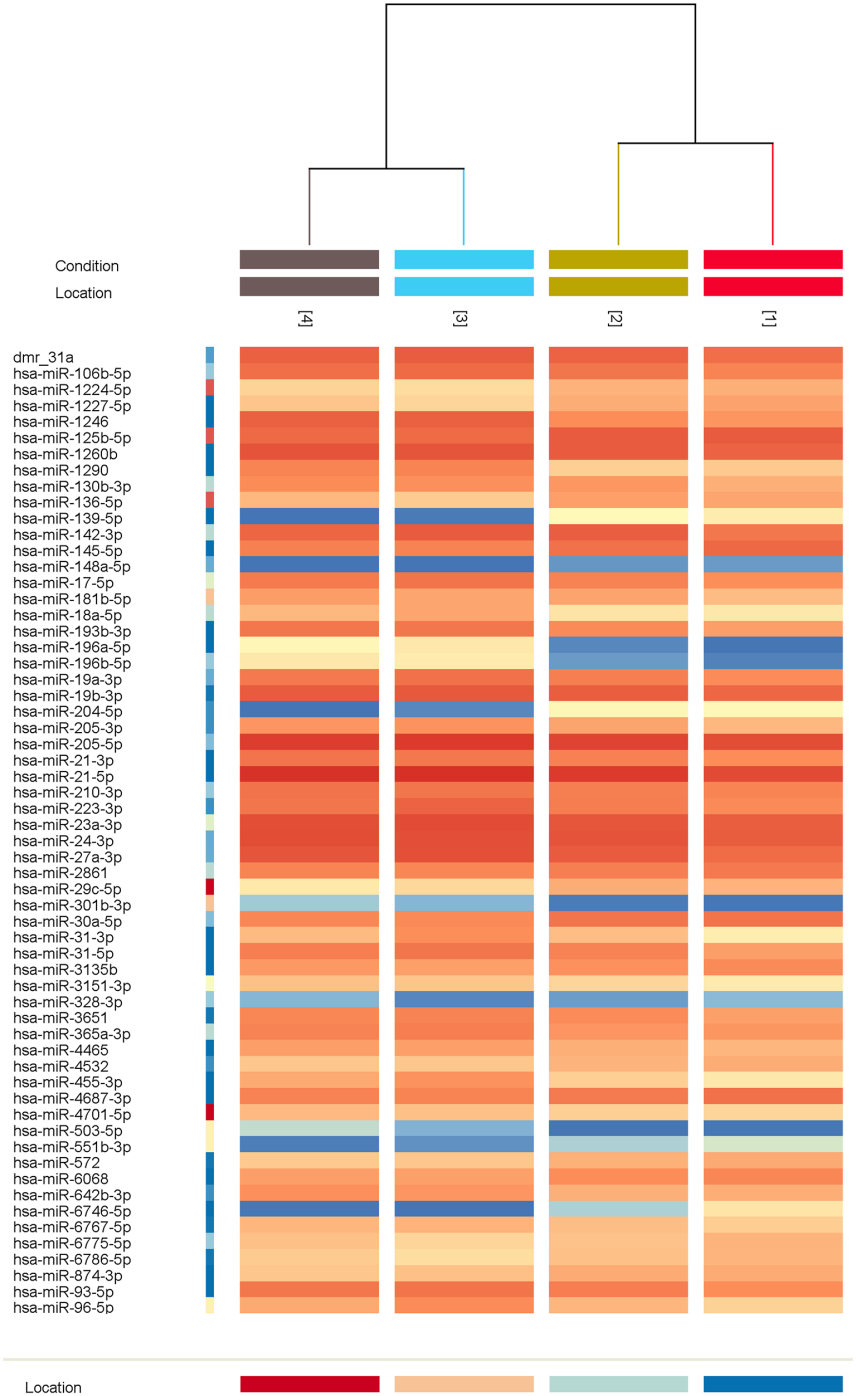
Cluster analysis of significantly dysregulated miRNAs from the global microarray profile of 2549 microRNAs in 12 patients with advanced laryngeal carcinoma^7^. Legend: (1) tumor surface; (2) tumor depth; (3) peritumor mucosa; (4) normal laryngeal mucosa.

Samples from twelve patients were analyzed via a microarray technique for all 2549 microRNAs. After quality control procedures and normalization, the Friedman test (adjusted with Bonferroni correction) was used to determine statistically significant differences in microRNA expression levels. Fifty-nine microRNAs were found to have significantly different expression levels, as 37 were upregulated in comparison to paired normal laryngeal mucosa and 22 were downregulated at both the tumor surface and depth (Fig. 1). Ten microRNAs showed significantly different expression between sample groups (*p* < 0.05) and exhibited significant dysregulation in peritumor mucosa, consistent with the pattern of dysregulation in tumor samples—miR-1260b, miR-31-3p, miR-31-5p, miR-93-5p, miR-21-3p, miR-181b-5p, miR-130-3p, miR-145-5p, miR-4687-3p, miR-6786-5p [Supplement 1].

### RT-PCR validation confirmed significant dysregulation of all twelve selected microRNAs in laryngeal carcinoma tumor tissue samples

All ten dysregulated microRNAs were validated with RT-PCR in all 240 samples (4 samples per case, 60 patients), along with two additional microRNAs—miR-210-3p and miR-144-3p. The latter were additionally chosen based on preliminary unpublished data of our own that revealed similar patterns of dysregulated expression, despite the lack of a confirmative microarray background. All twelve studied microRNAs showed significant dysregulation, as shown in Table 1. Two of those, miR-4687-3p and miR-6786-5p, we report for the first time in the literature to have altered expression in laryngeal cancer.

**Table 1:** Dysregulation rate among cases for all 12 validated microRNAs.

Up-regulated miRs	Overexpression in peritumoral mucosa (RQ > 2) (%)	Overexpression in tumor surface (RQ > 2) (%)	Overexpression in tumor depth (RQ > 2) (%)
miR 1260b	16.39	21.31	32.79
miR 130b-3p	18.03	49.18	49.18
miR 181b-5p	19.67	47.54	54.10
miR 21-3p	34.43	47.54	52.46
miR 210-3p	16.39	43.70	47.54
miR 31-3p	36.07	55.74	52.46
miR 31-5p	32.79	37.70	47.54
miR 4687-3p	21.31	40.98	42.62
miR 6786-5p	24.59	32.79	37.70
miR-93-5p	21.31	47.54	42.62

Down-regulated miRs	Downregulation in peritumoral mucosa (RQ < 0.5) (%)	Downregulation in tumor surface (RQ < 0.5) (%)	Downregulation in tumor depth (RQ < 0.5) (%)
miR 144-3p	72.13	65.57	67.21
miR 145-5p	68.85	77.05	72.13

### MiR-181b-5p shows significant heterogeneity in expression levels between tumor depth and surface

**Figure 2:**
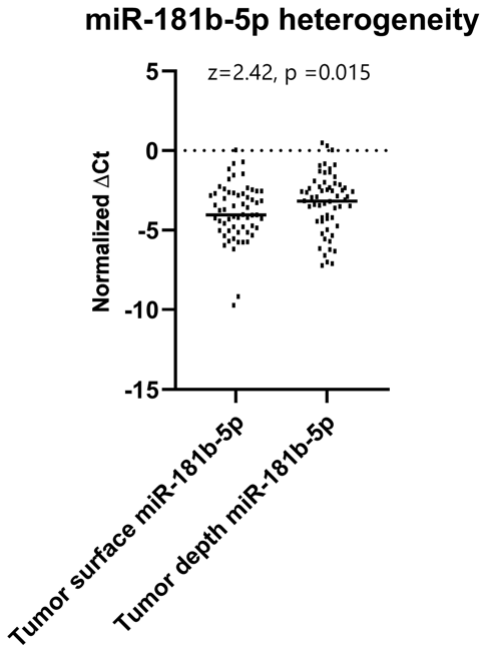
Significant difference in expression levels of miR-181b-5p between tumor surface and depth (pairwise comparison, Wilcoxon signed-rank test, z = 2.42, *p* = 0.015).

Subsequently, we analyzed the heterogeneity of the tumor tissue in our group of patients by performing a pairwise comparison between samples taken from the surface of the tumor and its depth. The expression levels of miR-181b-5p were significantly higher at the tumor depth than at the tumor surface (z = 2.42, *p* = 0.015) (Fig. 2). We could conclude from our data that tumor depth exhibits to some degree a more pronounced dysregulation pattern in the expression levels of the studied microRNAs in comparison to the tumor surface, but generally, there are no remarkable variations in terms of heterogeneity of the tumor tissue, except for miR-181b-5p.

### RT-qPCR validation confirms that tumor suppressors miR-144-3p and miR-145-5p were significantly dysregulated in peritumor mucosa with a pattern of expression consistent with that in paired tumor samples

**Figure 3:**
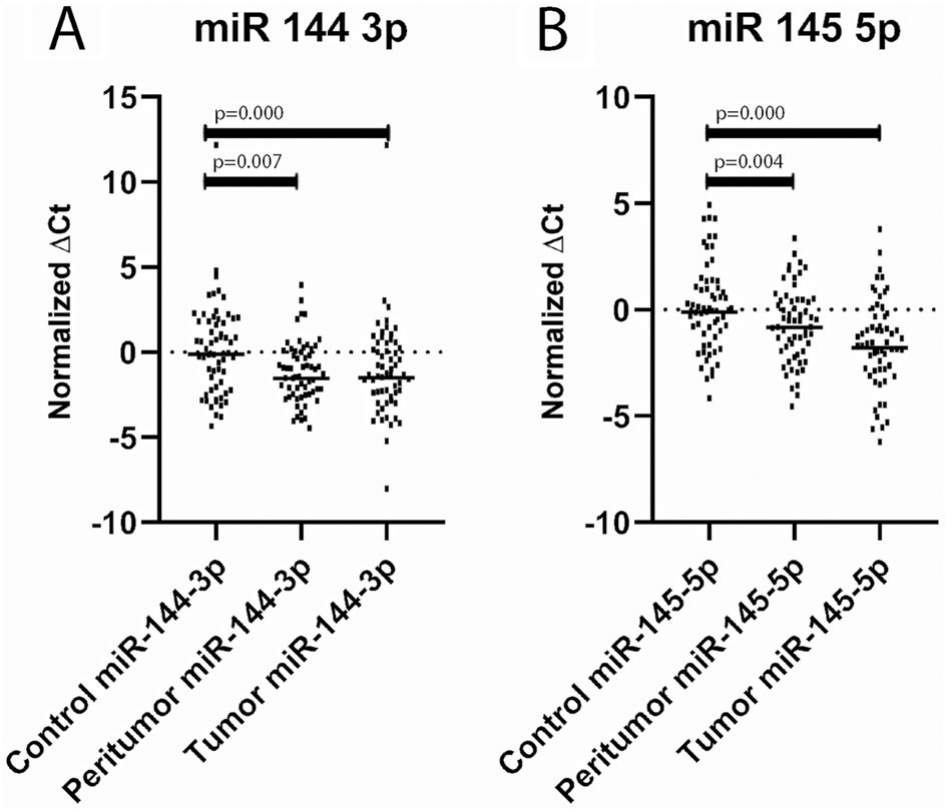
Statistically significant differences in the expression levels of miR-144-3p and miR 145-5p between tumor tissue, peritumor mucosa and healthy control laryngeal mucosa. Pairwise comparison using the Friedman test (with Bonferroni correction); miR-144-3p [mean rank]: 1.58 versus 2.14 versus 2.29, *p* = 0.000; miR-145-5p [mean rank]: 1.57 versus 2.15 versus 2.28, *p* = 0.000.

Pairwise comparison (with Bonferroni correction) between samples by using the Friedman test revealed that the two tumor suppressor genes miR-144-3p (mean rank 1.58 vs 2.14 vs 2.29, *p* = 0.000) and miR-145-5p (mean rank 1.57 vs 2.15 vs 2.28, *p* = 0.000) exhibited substantial expression dysregulation in peritumor mucosa, which corresponds with the pattern of dysregulation in tumor samples. Both microRNAs were significantly downregulated in peritumor mucosa similarly to tumor tissue (Fig. 3). The results from the microarray for the other ten oncogenic microRNAs regarding the peritumor samples were not confirmed by RT-PCR, and evidently, their expression in peritumor tissue does not reach significantly different expression levels from that in healthy control laryngeal mucosa. Survival analysis did not show any significant prediction for recurrence in relevance to the expression levels of these microRNAs in the altered peritumor mucosa.

### The expression levels of miR-93-5p, miR-144-3p, and miR-210-3p in tumor samples are strong predictors of recurrence-free survival in laryngeal carcinoma. MiR-144-3p exhibits a paradoxical association with survival

**Figure 4:**
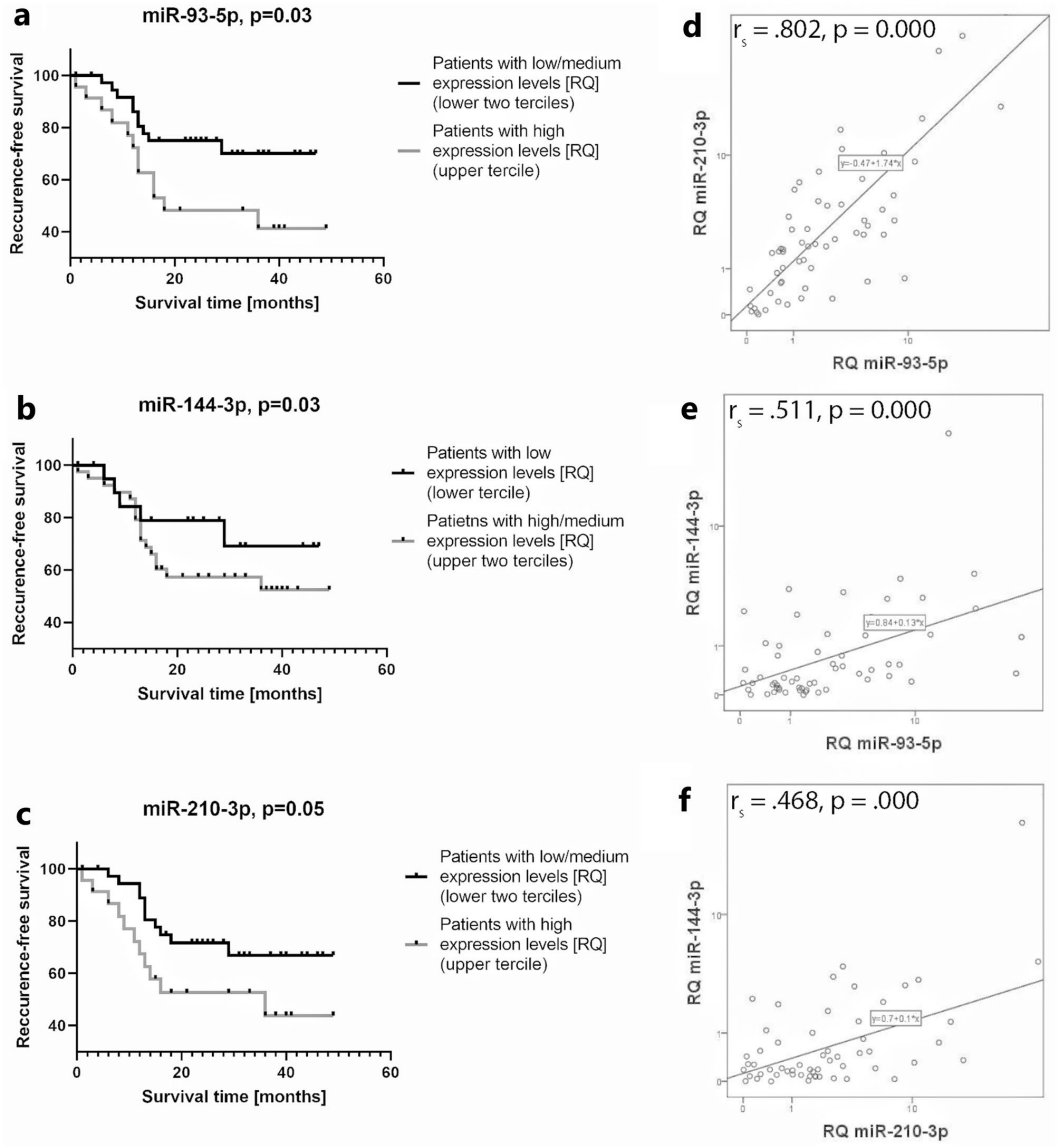
**(a–c)**: Kaplan–Meier curves illustrating that patients expressing higher levels of miR-93-5p, miR-210-3p and miR-144-3p had significantly worse survival rates than patients with lower expression levels (χ^2^(2) = 4.68, log-rank *p* = .03; χ^2^(2) = 4.53, log-rank *p* = .03, χ^2^(2) = 4.53, log-rank *p* = .03, respectively). (**d–f)** Spearman’s rank-order correlation reveals strong associations between miR-93-5p, miR-210-3p and miR-144-3p.

Recurrence-free survival was 63.9% for the whole study group. We divided patients into terciles based on RQ expression—low, medium, and high expression levels as in other similar studies^8^. The Kaplan–Meier method indicated that patients with high expression levels of the oncogenes miR-93-5p and miR-210-3p had significantly worse survival rates than patients with low/medium expression levels (χ^2^(2) = 4.68, log-rank *p* = 0.03; χ^2^(2) = 4.53, log-rank *p* = 0.03, respectively) (Fig. 4a,c). Breslow and Tarone-Ware tests also confirmed this finding (*p* < 0.05). The estimated median survival time in both the low/medium groups was not calculated since those patients did not reach 0.5 cumulative survival, but the median survival times for patients with high expression levels of miR-93-5p and miR-210-3p were 18 and 36 months, respectively. Additionally, when performing the same survival analysis with different cutoff values, e.g., RQ > 2 (patients with overexpression) or division of each group at median RQ value, the results for both microRNAs again confirm their significance as predictors of recurrence.

Analysis of miR-144-3p tumor suppressor expression would reveal not only dysregulation in peritumor mucosa similar to that in the tumor tissue but also very strong predictor capacity. Despite being massively downregulated (RQ < 0.5 in 67.21% of cases), which is consistent with the nature of a tumor suppressor gene, miR-144-3p exhibited a paradoxical survival association: patients with high/medium expression levels had significantly worse survival rates than patients in the lower tertile (χ^2^(2) = 4.53, log-rank p = 0.03) (Fig. 4b). The median survival time for the high/medium expression group was 18 months, and the low expression group did not reach 0.5 cumulative survival and was not calculated. Again, Breslow and Tarone-Ware tests also confirmed this finding (*p* < 0.05), and dividing subgroups according to different cutoff values (high vs low/medium, RQ < 0.5 or median RQ) similarly resulted in significantly better outcomes for patients with lower levels of miR-144-3p expression. Such an association with survival is typical for an oncogene, and this paradox is further reviewed in the discussion section.

Cox regression univariate analysis also confirmed the predictive capacity of these three microRNAs—miR-93-5p (Exp(B) = 1.110, *p* = 0.000), miR-210-3p (Exp(B) = 1.031, *p* = 0.000) and miR-144-3p (Exp(B) = 1.059, *p* = 0.023). Multivariate analysis with all three microRNAs identified miR-93-5p as a significant predictor for survival (Exp(B) = 1.126, *p* = 0.003), but the other two miRs did not have significance as independent predictors, which is expected since all three correlate with each other, as seen below.

Spearman's rank-order correlation was run to assess the relationship between each of the three microRNAs. Preliminary analysis showed the relationships to be monotonic, as assessed by visual inspection of a scatterplot. MiR-93-5p and miR-210-3p exhibited a very strong statistically significant positive correlation, as seen in the scatterplot, r_s_ = 0.802, *p* = 0.000 (Fig. 4d). A strong association was also found between the expression levels of miR-93-5p and miR-144-3p, r_s_ = 0.511, *p* = 0.000 (Fig. 4e) and between miR-144-3p and miR-210-3p, r_s_ = 0.468, *p* = 0.000 (Fig. 4F).

### MiR-1260b, miR-21-3p, miR-31-3p and miR-31-5p are strongly associated with tumor grade

A Kruskal–Wallis test was conducted to determine if there were differences in RQ expression levels between different tumor grades. We report four microRNAs that are significantly associated with tumor grade: miR-1260b, miR-21-3p, miR-31-3p, and miR-31-5p. All of these exhibited a quantitative expression gradient, with RQ levels being the highest in G1 tumors and lowest in G3 tumors [Supplement 2]. The scores of the pairwise comparison were as follows: miR-1260b: G1-39.40 versus G2-27.49 versus G3-17.33 [mean rank], *p* = 0.03; miR-21-3p: G1-41.07 versus G2-27.00 versus G3-15.67 [mean rank], *p* = 0.008; miR-31-3p: G1-35.53 versus G2-29.51 versus G3-9.00 [mean rank], *p* = 0.048 and miR-31-5p: G1-37.87 versus G2-28.88 versus G3-8.67 [mean rank], *p* = 0.018. Similarly, the correlation between these four microRNAs was strong, with r-values varying between 0.498 and 0.880 (Table 2). Finally, survival analysis of tumor grade showed no significant differences between subgroups despite observing some general tendency of worse outcomes in lower grade patients.

**Table 2:** Correlations between microRNAs associated with tumor grade (miR-1260b, miR-21-3p, miR-31-3p and miR-31-5p).

	RQ miR-1260b	RQ miR-21-3p	RQ miR-31-3p	RQ miR-31-5p
**RQ miR-1260b**				
Corr. Coeff	1.000	0.742**	0.498**	0.606**
Sig. (2-tailed)	–	0.000	0.000	0.000
N	60	60	60	60
**RQ miR-21-3p**				
Corr. Coeff	0.742**	1.000	0.639**	0.694**
Sig. (2-tailed)	.000	–	0.000	0.000
N	60	60	60	60
**RQ miR-31-3p**				
Corr. Coeff	0.498**	0.639**	1.000	0.880**
Sig. (2-tailed)	0.000	0.000	–	0.000
N	60	60	60	60
**RQ miR-31-5p**				
Corr. Coeff	0.606**	0.694**	0.880**	1.000
Sig. (2-tailed)	0.000	0.000	0.000	–
	60	60	60	60

*Correlation is significant at the 0.05 level (2-tailed).

**Correlation is significant at the 0.01 level (2-tailed).

## Discussion

Microarray expression profiling has established itself as a powerful tool for quantitative expression analysis and for finding dysregulations among an extensive number of genes, but like every method, it has its pitfalls. First, we compared our profiling results with microarray microRNA data of laryngeal carcinoma samples published in the literature. We found nine articles that reported these miRNAs, and with the help of a Venn diagram, we summarized and visualized the distribution of our dysregulated microRNAs overlapping among different studies [Supplement 3]. This diagram undoubtedly represents the expected variations in the outcome that a single method could produce when several factors are variable. We found plausible explanations for discrepancies in three major directions: (1) different control groups—a significant number of studies use nonpaired smaller control sample groups, some of them even used samples from other anatomical subsites (e.g., esophageal mucosa); (2) smaller number of cases; (3) different degrees of homogeneity of the study groups in terms of mixing different subsites and stages of cancer. All microRNAs selected after microarray analysis in our study were confirmed to be dysregulated by RT-PCR. We report miR-6786-5p and miR-4687-3p for the first time in the literature to be deregulated in laryngeal carcinoma. There are a limited number of studies reporting dysregulation of these two genes in other malignancies^9,10^.

Peritumor mucosal microRNA dysregulation is a very recent research topic of interest, as it represents a new direction for investigating the classical concept of "field cancerization" proposed by Slaughter in 1953^11^. Two other research groups have published on this matter. Ganci et al. investigated peritumor tissue samples in patients with head and neck cancer and found that miR-429, miR-96-5p, miR-21-5p, and miR-21-3p were dysregulated in those samples and that their expression levels predicted recurrence in the study group^8^. Using only RT-PCR, Orosz et al. reported different dysregulation patterns of microRNA expression (miR-21, miR-27a, miR-34a, miR-143, and miR-146a) in peritumor mucosa from pharyngeal carcinomas, which differ depending on the subsite of the tumor^12^. The latter study did not include survival analysis. Three out of the four microRNAs in Ganci’s study (all except miR-429) were also registered as dysregulated in our microarray profile, and only two of the five microRNAs in Orozs’s study (miR-21, miR -27) overlapped with ours. Both studies included cohorts of patients with different subsites of cancer origin in the area of the head and neck. As seen in Orosz’s study, different locations have different patterns of dysregulated microRNAs, and this factor could alone explain the differences in miRNA profiles reported in the literature. Additionally, the inclusion of different stages could also be a factor, as seen in other reports^13^. In our study, we focused on advanced-stage laryngeal cancer (87.1% T4a) to achieve maximum homogeneity of the group. Moreover, we analyzed two samples from each tumor (surface and depth) to uncover potential tumor heterogeneity. Our data showed that in the peritumor mucosa, the tumor suppressors miR-144 and miR-145 from the microRNA validation group were significantly dysregulated compared to the paired normal mucosa in a pattern identical to tumor samples. This finding supports the field cancerization theory that on the molecular level, some processes of cell dysregulation characteristic of malignancy are evident in clonal patches of the surrounding mucosa, and presumably, both have a common predecessor. Survival analysis did not show a predictive value of the expression levels of these two microRNAs in peritumor mucosa despite having one of them as a predictor in tumor tissue (miR-144). An explanation could be found in the specifics of surgical treatment—in advanced laryngeal cancer due to the removal of the whole organ, the surgeon would resect far greater resection margins in comparison to other head and neck cancer sites such as pharyngeal lesions where standard free-margin resection is generally done. Due to this specificity, these patches of field cancerization in laryngeal carcinoma are often resected (sometimes unconsciously), and the stochastic chance of local recurrence due to field cancerization is significantly lower. These wide patches or gradients of dysregulated mucosa far beyond the tumor borders have been well described and visualized by Orosz in another study of oropharyngeal carcinoma^14^.

Survival analysis of our data undoubtedly points out miR-93-5p, miR-144-3p, and miR-210-3p as strong predictors for recurrence in laryngeal carcinoma. Three other microarray studies on laryngeal carcinoma reported that miR-93-5p was also dysregulated^15–17^, as two of these validated it with RT-PCR^16,17^. MiR-210-3p has been identified only in one microarray profile that used laryngeal cancer cell lines^18^, and miR-144-3p has been only recently outlined as a downregulated gene in laryngeal carcinoma in a paper dedicated solely to this microRNA by using available data in public databases^19^. However, we are the first to report them as clinical predictors of recurrence in advanced laryngeal carcinoma. While miR-93-5p and miR-210-3p are known oncogenes and their overexpression corresponds with a higher chance of recurrence, we found an intriguing association of the expression levels of miR-144-3p with survival. Despite being massively downregulated in tumor tissue, which corresponds with the nature of a dysregulated tumor suppressor gene, higher expression levels of miR-144-3p in our data exhibit a very strong association with a higher probability of recurrence. MiR-144-3p is a known tumor suppressor^20^, but this paradoxical positive correlation is typical for oncogenes. We could easily conclude that it has oncogenic potential, but this would not correspond to the general downregulation in the study group. In an attempt to find an explanation for this paradox, we discovered a very interesting number of studies that established a connection between some downregulated genes and copy number aberrations (CNAs). Significantly downregulated genes have often been found to reside within chromosomal regions with an increased number of copies (gains) and vice versa, creating a paradoxical signal^21^. Phillips et al.^22^ reported that 14% of the genes downregulated in prostate cancer reside within regions of DNA copy number gains, and approximately 9% of upregulated ones reside in regions of DNA copy number loss. We hypothesize that different numbers of DNA copies of the region coding miR-144-3p could be the reason for the small discrimination differences in its expression levels, and these small changes in RQ could strongly correlate as a marker for the expression of one or more linked genes in the same DNA region and thus explain its strong paradoxical association with survival. The controversial oncogene behavior of miR-144-3p has been previously reported in some other solid tumors, such as nasopharyngeal^23^ and papillary thyroid carcinoma^24^, although in the majority of solid tumors, it is downregulated^25^. MiR-144-3p is a key regulator that has an effect on many downstream effector genes and thus participates in the regulation of key signaling pathways and tumor biology; however, differences between tissues could also be observed.

Correlation analysis provides an additional overview of these processes—the group of microRNAs that have a predictive value (miR-93-5p, miR-144-3p, and miR-210-3p) have strong correlations with each other. This finding suggests that they have a common pathological synergy and regulatory framework, and as multivariate analysis shows, they are not independent. The other group of microRNAs (miR-1260b, miR-21-3p, miR-31-3p, and miR-31-5p) also exhibited a strong correlation with each other and were statistically associated with tumor grade. Since survival according to our data is not significantly associated with tumor grade, these four biomarkers are not expected to demonstrate predictive benefit. In other studies, conflicting survival data could be found, e.g., miR-21-3p could be a strong marker for survival^23,26^, and the same applies to tumor grade^27,28^. A lower tumor grade is associated with a higher rate of local metastasis; thus, thorough lymph node dissection is compulsory for lower rates of local–regional failure^29^. Some studies include cases treated with different surgical protocols, and this could influence metastatic recurrences, which would on the other hand validate microRNAs that are associated with tumor grading as markers for survival. Additionally, the expression levels of some of those miRs are proportional to tumor stage, and the inclusion of such heterogeneity also strongly influences survival outcomes among subgroups.

**Figure 5:**
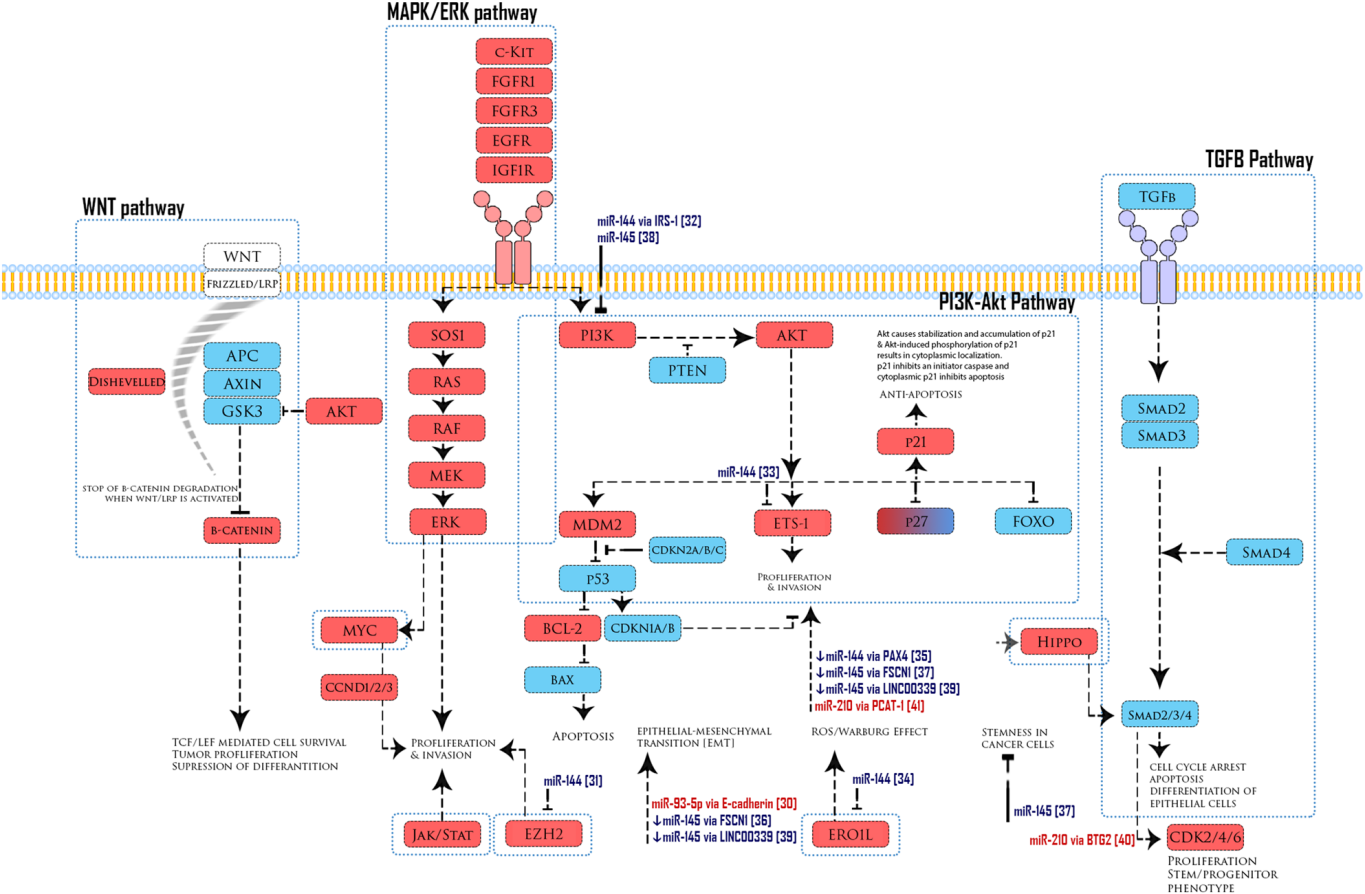
Illustration of some major cascades in cancer with a summary of all validated targets of miR-93-5p, miR-210-3p, miR-144-3p, and miR 145-5p in laryngeal carcinoma published in the literature. Blue color designates tumor suppressor genes, red color—oncogenes and references are given in brackets.

Finally, we summarized all published and verified targets of miR-93-5p, miR-210-3p, miR-144-3p, and miR 145-5p in laryngeal carcinoma in the literature and illustrated those findings along with all major cancer cascades (Fig. 5).

## Conclusion

To our knowledge, this is the first comprehensive microarray profiling of advanced laryngeal carcinoma with such a degree of homogeneity of the study group in terms of tumor stage and site, unified surgical and treatment protocol (single surgeon consecutive series), with paired control samples. A specific microRNA signature of dysregulation in tumor samples, as well as in histologically healthy peritumor laryngeal mucosa, was identified and successfully validated by RT-PCR. Of all twelve miRNAs with significantly changed expression, miR-4687-3p and miR-6786-5p are reported for the first time in laryngeal carcinoma. We provide new insights into field cancerization theory in laryngeal carcinoma by showing two tumor suppressors, miR 144-3p and miR 145-5p, to be significantly downregulated in peritumor mucosa, similar to tumor tissue dysregulation. Based on our results, three strong biomarkers for the prediction of recurrence of this malignancy are proposed: miR-93-5p, miR-144-3p, and miR-210-3p. Additionally, we outline a set of microRNAs that are associated with tumor grade and review their possible impact on survival depending on treatment standardization.

In terms of future lines of work, it is important to translate these findings into clinical perspective. Recent advances in in situ hybridization technology could be used to visualize field cancerization and possibly this could be a chance for shifting the paradigm from histological tumor-free margins to molecular-healthy resection lines thus achieving better oncological outcomes.

## Materials and methods

### Study design and treatment protocols

Sixty patients (mean age at diagnosis was 64.6 with a standard deviation of 8.7 years) with pathologically verified primary laryngeal carcinoma were enrolled in the current prospective prognostic study. All patients underwent primary laryngectomy in 2018–2019 at the Department of ENT, Head & Neck Surgery, Medical University—Sofia. During surgery four samples from each patient were obtained: two from the tumor site –surface and depth, 3rd sample was taken from histologically healthy peritumor mucosa within 1 cm from the border of the tumor and the 4th sample was paired normal laryngeal mucosa distant to the tumor (contralateral, at least 3 cm distance). All samples were stored in RNAlater Solutions (Thermo Fisher Scientific, Massachusetts, USA) and frozen at − 20 °C for a short period. Collected samples were transported to the Molecular Medicine Center, Department of Medical Chemistry and Biochemistry, Medical University—Sofia, and maintained at−80 °C until use. The study was approved by the Ethical Committee of Medical University—Sofia, and written informed consent was signed by every patient. The enrolled cohort was a single-surgeon consecutive series and the inclusion criteria were advanced-stage laryngeal squamous cell carcinoma (T3 or T4 stage). Surgical treatment included laryngectomy with free resection margins and neck dissection ipsilaterally (2–5 levels). In cases of tumors crossing the median line, contralateral neck dissection was also performed (2–5 levels). Additionally, if the tumor extended into the subglottic or retrocricoid region, a full paratracheal lymph node dissection (levels 6–7) was carried out. All patients underwent postoperative radiotherapy or combined chemoradiotherapy according to the protocol.

The follow-up period was an average of 24 months with a standard deviation of 13 months. Patients were followed-up every month during the first 6 months after surgery and every 3 months after this period. Every 6 months, PET-CT was scheduled for radiological evaluation. Cases were registered as events when recurrence or death due to malignancy was found. Censoring was recorded when death due to other causes not connected to the primary disease existed. A notable comment should be made about the fact that five of the patients were censored due to COVID-19-related death with no signs of recurrence, which significantly decreased the average follow-up period.

### RNA extraction

Total RNA (including miRNAs) was isolated from 60 fresh-frozen tumor materials and adjacent normal tissue using the miRNeasy Mini Kit (Qiagen, Hilden, Germany) according to the manufacturer’s recommendations. The quantity of the RNA was assessed using a NanoDrop 2000 spectrophotometer (Thermo Fisher Scientific, Massachusetts, USA). The Qubit™ RNA HS Assay Kit, Qubit™ 2.0 Fluorometer (Life Technologies, Thermo Fisher Scientific Inc.) was used for precise quantification of 50 ng/µl and 100 ng/µl RNA dilutions used further for miRNA array and RT-qPCR analysis, respectively. RNA integrity numbers (RIN) were evaluated with a 2100 Bioanalyzer (Agilent Technologies, California, USA) according to the manufacturer’s recommendations, and samples with RIN > 5 were used in the array studies.

### miRNA expression microarray profiling

MicroRNA microarrays from Agilent Technologies (G3 Human MiRNA Microarray Kit, Release 21, 8 × 60 K) with AMADID No. 070,156 were used for microRNA expression profiling. The procedures for conducting this study were described in a previous publication by the group^7^.

### Real-time quantitative polymerase chain reaction (RT-qPCR)

Complementary DNA (cDNA) was synthesized from 400 ng of total RNA using the miScript II RT Kit (Qiagen, Hilden, Germany) according to the manufacturer’s recommendations. The primers for the selected mature miRNAs were designed by Qiagen as miScript Primer assays (Qiagen, Hilden, Germany) as follows: miR-21-3p; miR-31-3p, miR-31-5p, miR-93-5p, miR-130b-3p, miR-144-3p, miR-145-5p, miR-181b-5p, miR-210-3p, miR-1260b, miR-4687-3p and miR-6786-5p. RT-qPCR was performed by using a miScript SYBR Green PCR kit (Qiagen, Hilden, Germany) on a 7900HT Fast Real-Time PCR System (Applied Biosystems, California, USA). Each sample was analyzed in triplicate with a total volume of 10 μL. The mean values of Ct from the three repeats were used for the data analysis, and the amounts of the PCR products were normalized to U6 (Qiagen, Hilden, Germany), which was used as an internal control. Negative and no-template controls were also evaluated. The relative quantification (RQ) of miRNAs in samples was analyzed by the 2-ΔΔCt method, as previously described^6,42^. RQ over 2.00 was defined as overexpression, and RQ less than 0.5 was defined as underexpression.

### Statistical analysis

Data analysis was performed with SPSS software ver 23.0 for Windows (IBM SPSS, NY, USA) and GraphPad Prism software. The Kolmogorov–Smirnov test for normality, Wilcoxon test, Mann–Whitney U test, Kruskal–Wallis or one-way ANOVA test, and paired and unpaired T-tests were used as appropriate. The relations between two continuous variables were evaluated using the bivariate correlation coefficient Spearman’s rank test. Correlations between the expression levels of miRNAs and clinicopathological features were analyzed using Kruskal–Wallis rank tests for k independent samples and the Mann–Whitney U test for independent association analysis between any two subgroups. The Friedman test was used to determine differences between the tumor, peritumor mucosa, and control laryngeal mucosa. Recurrence-free survival was calculated from the date of diagnosis until recurrence or death caused by the malignancy was registered. Survival curves were plotted through the Kaplan–Meier method, and the log-rank test was used to compare survival between groups. Additionally, univariate and multivariate Cox regression analyses were performed to test the association of various factors with survival time. A two-tailed *p*-value ≤ 0.05 was considered significant.

### Ethics approval

The Ethical Committee of Medical University—Sofia approved the study and written informed consent was signed by every patient. The study was performed in accordance with the Declaration of Helsinki.

## Supplementary Information

Supplementary Information 1.

Supplementary Information 2.

Supplementary Information 3.

Supplementary Information 4.

Supplementary Information 5.

## Data Availability

Raw data were generated at Medical University—Sofia. Derived data supporting the findings of this study are available from the corresponding author [TP] on request. Raw data is added as supplement 4.
