# Supplementary figures and images for: Global microRNA expression profile in laryngeal carcinoma unveils new prognostic biomarkers and novel insights into field cancerization
Source: Sci Rep. 2022 Oct 12;12:17051. doi: 10.1038/s41598-022-20338-w (PMC9556831; doi:10.1038/s41598-022-20338-w)

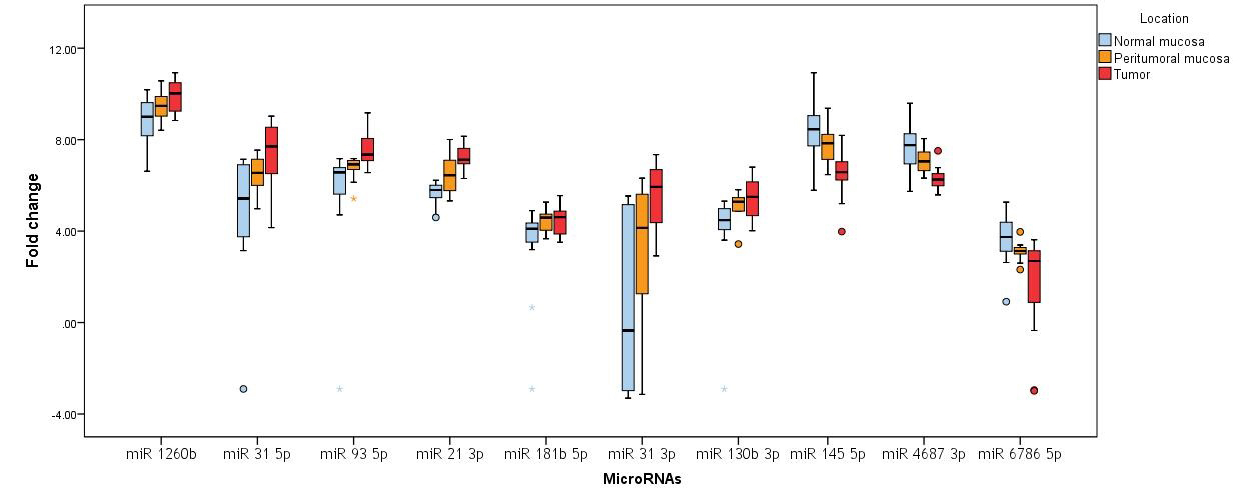

Supplement: Supplementary file 1 — Supplementary Information 1. [file 41598_2022_20338_MOESM1_ESM.tif]

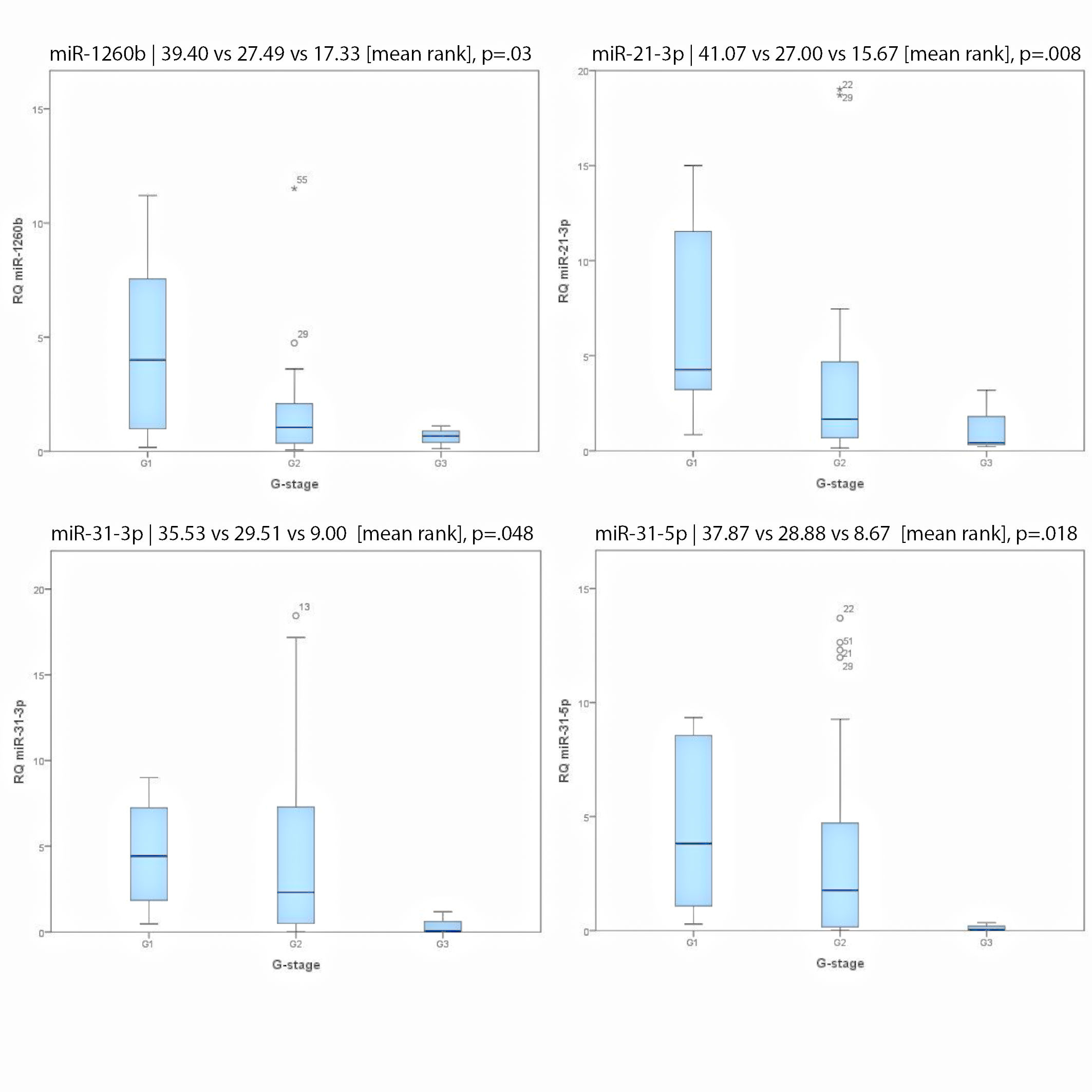

Supplement: Supplementary file 2 — Supplementary Information 2. [file 41598_2022_20338_MOESM2_ESM.tif]

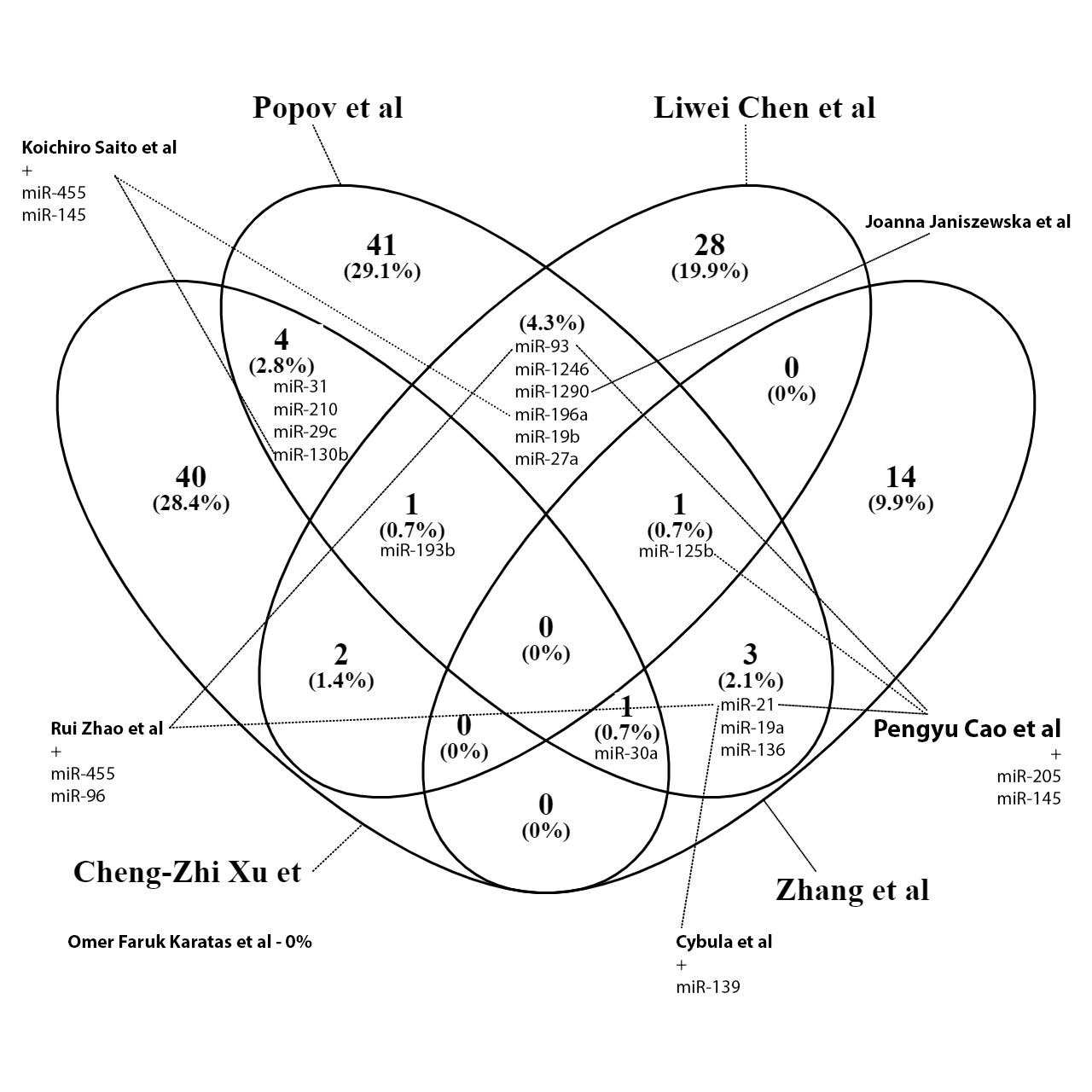

Supplement: Supplementary file 3 — Supplementary Information 3. [file 41598_2022_20338_MOESM3_ESM.png]
