# Supplementary material for: Global microRNA expression profile in laryngeal carcinoma unveils new prognostic biomarkers and novel insights into field cancerization
Source: Sci Rep. 2022 Oct 12;12:17051. doi: 10.1038/s41598-022-20338-w (PMC9556831; doi:10.1038/s41598-022-20338-w)
Supplement: Supplementary file 4 — Supplementary Information 4. [file 41598_2022_20338_MOESM4_ESM.pdf]

BOXPLOT

Sample	normalized miR-144-3p	normalized miR-145-5p	normalized miR-181b-5p	normalized miR-210-3p	normalized miR-21-3p	normalized miR-31-3p
i1.1	3.43205175	1.246004669	2.950649136	1.419650147	1.731626164	2.507675714
i1.2	-1.0209189	0.215997003	-2.51672933	0.473657481	0.743809497	-8.66843267
i1.3	1.24649442	-0.018258	-0.96566567	2.858654147	2.973108164	-6.13973733
i1.4	1.15046842	0.510521003	-1.29232167	3.888673814	3.041196164	-5.60220867
i2.1	-0.4959266	1.200237669	-3.500344	-0.67879285	-0.85106284	-8.31820167
i2.2	-0.3705106	0.605715003	-3.50536667	-0.30208085	-0.4410345	-8.36193733
i2.3	-1.4240022	-3.01267133	-3.58083667	1.604548814	1.100506831	-7.427529
i2.4	1.07449792	-2.44551833	-3.042288	2.486670147	1.377181831	-5.82945433
i3.1	-2.4135182	-0.06720333	-2.99891	-0.52395285	-0.1088305	-9.18335733
i3.2	-1.6906776	-0.60869133	-3.982137	-0.60879152	-0.1769815	-7.97891267
i3.3	-1.7814102	-3.65477733	-3.16028967	-1.00552419	-1.08286084	-7.28521933
i3.4	-3.2878297	-1.08706333	-1.08963333	0.697407814	0.629498164	-8.27450767
i4.1	-1.9532962	-0.30468366	-3.73555867	0.248749147	-0.42393317	-9.74999533
i4.2	-1.0529065	-2.611485	-3.845506	-1.44406752	-0.81517417	-9.87632833
i4.3	0.55828875	1.382383003	-1.60441467	2.498559481	1.944065497	-6.88588967
i4.4	-3.8737564	-2.331845	-1.53191733	0.547906147	0.536504497	-12.5934043
i5.1	3.25674942	4.182983336	-0.02606867	6.143914814	3.678060831	-6.12345733
i5.2	2.04239108	1.594320669	-0.88683833	2.578003814	2.334873164	-7.340857
i5.3	-1.5092319	-1.034558	-2.863758	1.410404147	1.509132831	-8.552937
i5.4	-0.4576649	-0.26683266	-2.20244567	2.803948481	1.460276497	-7.82755367
i6.1	0.99652842	0.147105669	-3.022199	1.168133814	0.720329831	-7.38987833
i6.2	-1.6401292	0.314861669	-3.20561767	0.817761814	0.874121831	-7.00948867
i6.3	-0.9416212	-2.019376	-2.980232	3.115020147	1.118339164	-4.925257
i6.4	-1.7666242	-2.15564866	-2.482658	1.932070814	0.372773831	-5.07867933
i7.1	1.91504375	4.779178003	0.023567	4.878722981	2.619219164	-6.45825667
i7.2	1.76927175	1.018657336	-1.800035	2.258951481	0.368570497	-6.42202567
i7.3	-2.5673332	-2.222026	-3.514379	-0.37479202	-0.3805525	-10.6533137
i7.4	-0.5629752	3.23361E-05	-2.42440433	-2.38702119	0.542688831	-11.1818597
i8.1	4.60359508	4.138922336	-0.36276533	5.112723314	2.637610164	-4.95072667
i8.2	-4.2388642	-1.05798733	-3.25509267	0.584229981	-1.2650235	-8.83817033
i8.3	-4.1498736	-0.32034933	-1.69990733	-2.25866285	1.594546497	-4.29312967
i8.4	-3.7282859	-1.626471	-3.00371533	0.240240814	-0.1364435	-5.80454467
i9.1	4.28160575	4.118133336	-1.654542	1.299261814	1.242187831	-8.72737367
i9.2	0.19541308	1.926280336	-1.90489433	1.209755481	0.779394164	-7.15124567
i9.3	0.40622008	0.752071003	-2.96473367	0.374468481	1.626271497	-7.33062067
i9.4	-0.6237016	3.314755336	0.336226333	4.219765481	4.256724164	-6.57973033
i10.1	3.06027442	1.979162336	-2.650374	1.766276147	-0.3363095	-8.535555
i10.2	0.39073408	-0.50597033	-3.49568467	3.647952147	0.915489831	-7.280439
i10.3	-2.7939929	-1.12901433	-2.48819533	2.135570814	-0.10696984	-8.241649
i10.4	-3.4137136	-0.54351	-2.05250533	2.378863147	0.376881164	-7.723412
i11.1	0.69408508	3.315933669	-2.51405567	2.493425481	0.651478497	-8.25703867
i11.2	-2.7044299	1.838813003	-3.28148833	0.607876814	-0.62489617	-8.90033133
i11.3	-1.5663142	-0.46260166	-2.401124	3.449713481	1.584382497	-4.84749633
i11.4	-2.5860349	-0.907745	-3.66499733	2.522308481	0.530156497	-5.99168933

i12.1	0.00201508	2.194899003	-2.87978367	2.223937481	0.264141831	-7.528187
i12.2	0.25877142	0.282544003	-2.72982433	0.267771814	0.606590831	-6.637981
i12.3	-1.7692392	-0.97443733	-2.55796733	0.727046147	1.087172164	-6.49024533
i12.4	0.01427642	0.384827669	0.154153667	2.967536481	2.324192831	-5.43510867
i13.1	-1.3287219	3.314269669	-2.259322	3.109677481	0.178107831	-9.45201433
i13.2	-3.9818766	-1.00886133	-3.586688	-1.31649752	-1.52950917	-10.7738172
i13.3	-0.6120216	-0.07435466	-2.84620067	1.934521814	2.145701164	-6.528664
i13.4	-3.2012557	0.169558003	-2.64504333	0.520938147	0.420183164	-5.24704133
i14.1	-0.2392346	-0.27693866	-6.95201667	-2.03651919	-2.46840517	-12.5949263
i14.2	-2.3385239	-2.08701033	-6.76143033	-1.69252019	-0.6873325	-10.635176
i14.3	0.88408942	-0.45112566	-1.941715	4.237132481	4.718754497	-6.274766
i14.4	1.02833675	-0.6988	-2.49074067	4.530127147	4.718286164	-7.25170533
i15.1	-3.0596344	-2.8871215	-8.19094117	-4.80006435	-3.808898	-14.6703118
i15.2	-4.0681555	-0.95567591	-7.24648558	-2.96388244	-1.93977742	-14.1836239
i15.3	-3.1773322	-1.679728	-5.89345667	0.186447481	0.994911164	-9.32924533
i15.4	-4.7575445	-1.99942721	-7.25422154	-1.43039473	-1.02633271	-12.6774159
i16.1	-2.8876702	-2.99547433	-6.11275367	-0.37026185	-0.07258484	-10.7884447
i16.2	-3.7402726	-3.09456366	-6.780117	-1.45691785	0.158230164	-10.5323823
i16.3	-1.0336109	-4.61261533	-5.90646867	-0.97698052	1.097497164	-14.6598337
i16.4	-3.7492809	-5.148497	-5.85599033	-2.23765752	-0.0192975	-14.9113633
i17.1	-3.0181342	-3.38800466	-7.08320033	-0.29946585	-0.4148195	-10.8823403
i17.2	-3.3893499	-2.864107	-5.90179967	-1.95378185	-0.28165417	-9.932698
i17.3	-1.1523149	-2.93742166	-5.83981733	0.724997147	2.180022831	-9.14388667
i17.4	-1.4779496	-1.57088233	-6.48022667	1.644725481	2.585474497	-7.71125667
i18.1	-1.6518662	-1.13523	-6.217848	-1.53308552	-0.4971315	-10.5771133
i18.2	-2.9419602	-3.70825966	-6.236834	-2.22527319	0.312345164	-11.548979
i18.3	-2.4244586	-2.78512233	-4.87919267	-0.55172919	-0.34906384	-11.7450313
i18.4	-3.1663139	-3.044916	-5.70728633	-0.25801652	1.180224164	-14.9172987
i19.1	-2.7055429	-2.276554	-5.69497867	-0.87908819	0.890662164	-10.231689
i19.2	-2.9197962	-2.114143	-6.72033467	-2.31859719	-0.43505917	-12.2695303
i19.3	-2.5458029	-3.27965866	-3.907175	-0.55224019	1.744196497	-13.4816883
i19.4	-2.2976746	-3.255402	-3.30714967	1.862128814	1.424686497	-13.726708
i20.1	-0.6097206	-2.13475433	-6.42146367	0.610950147	0.252050497	-12.207506
i20.2	-2.2767719	-2.6659475	-6.25582517	-0.05972685	-0.37698817	-10.9589907
i20.3	-3.4919866	-3.536363	-5.471587	-0.53848519	0.480060497	-10.1873807
i20.4	-3.8147496	-4.69004366	-5.131473	-1.22040085	-0.05186484	-12.6391923
i21.1	-2.4093746	-2.15385966	-7.15506267	-1.44636685	-0.22684884	-12.808976
i21.2	-1.0871866	-0.37597166	-4.40744667	1.275771147	1.441244497	-11.372433
i21.3	-3.0265642	-1.14504333	-5.91083167	-1.40366552	-0.11025917	-11.6324387
i21.4	-3.5443252	-1.12935766	-3.54642133	-0.36824719	2.503481831	-8.70734333
i22.1	-0.7886849	-0.06979633	-6.610795	-1.57893652	-0.3398165	-12.693154
i22.2	-0.9654696	1.722971336	-2.82322767	-0.07231952	2.483410164	-9.00165233
i22.3	-4.2419199	1.741848003	-4.35692567	-0.61355352	1.624797164	-11.7294923
i22.4	-0.3535556	3.334042669	-1.434483	2.594422147	3.886620831	-7.77528533
i23.1	1.63002875	-0.188343	-5.85174	0.770786814	0.643297831	-11.0184477
i23.2	-1.7928386	-4.707217	-6.51521767	-2.91151752	-0.0894865	-14.361071
i23.3	-2.4619469	-4.62271433	-4.943229	-1.89792919	-2.62796284	-13.613439

i23.4	-0.6385186	-3.04313433	-4.885056	0.316434814	-0.85018184	-11.9959427
i24.1	-1.1841029	-0.898082	-6.09675233	-0.65787985	-1.62606717	-11.4022237
i24.2	-2.8431482	-1.557081	-7.000908	-3.13006052	-1.32281284	-12.620427
i24.3	-3.1677989	0.874232669	-1.30549767	1.693466147	3.376358497	-12.4180033
i24.4	-1.4367069	-1.026338	-3.34215067	1.512934481	0.968599164	-12.442051
i26.1	-0.3173269	-0.940286	-7.02759467	-0.71842685	-1.5274265	-11.3776037
i26.2	-2.2296396	-1.12805533	-6.85549467	-3.73504885	-2.16491217	-13.865124
i26.3	-2.0137946	-1.74506766	-5.167024	1.722431147	-0.00391184	-10.1498287
i26.4	-2.7247452	-2.002106	-6.31005	0.200884814	-0.20211617	-12.3178207
i27.1	-4.5384572	-0.82123966	-4.06191467	0.682611147	1.721612831	-7.97734433
i27.2	-0.1326482	-2.97949733	-6.138697	-1.16083619	-0.19409517	-10.1790923
i27.3	-1.8196582	-5.691396	-4.29987033	-0.86015119	-0.49815284	-12.3658703
i27.4	-3.5222636	-3.404278	-3.057963	0.051066814	0.494908164	-15.6513667
i28.1	-0.4441442	-1.86564766	-4.18865167	-0.39616085	-1.0884585	-9.148179
i28.2	-1.0482482	-2.280902	-4.12617867	-0.24751685	0.037233831	-7.52566567
i28.3	-0.2443382	-2.078992	-3.236223	-0.03763519	0.053189831	-6.47275333
i28.4	0.00710508	0.600612669	-1.04562733	1.655813814	2.819107164	-6.23585567
i30.1	2.05465008	-0.980648	-5.288827	-2.49048385	-2.6399285	-10.9597183
i30.2	0.00126075	-1.73226266	-4.62749133	-0.98183152	-1.3453845	-8.95433833
i30.3	2.85251442	-4.871549	-2.686233	-1.04984252	-0.85522984	-4.16095467
i30.4	0.56297375	-0.92141533	-0.97945467	1.952208481	1.609965164	-6.307143
i31.1	0.68236242	-0.61308633	-6.55656333	-2.02518252	-1.82811984	-14.1268407
i31.2	-2.2541339	-1.74503333	-5.78259733	-1.85681352	0.116400831	-10.0254243
i31.3	0.99070142	-2.125234	-5.01315333	0.611706814	1.282193164	-7.719284
i31.4	0.25779408	0.862673336	-1.788928	1.889871481	2.863611497	-6.45493167
i32.1	1.81232208	-0.43954633	-2.86501967	0.252334147	0.902972831	-8.89059767
i32.2	-0.0986179	-0.38584266	-2.92017	0.010214814	0.375284831	-8.69298333
i32.3	1.30237842	-5.44678466	-3.62593233	0.349657147	0.073477831	-12.784812
i32.4	-0.7324322	-4.05130566	-3.66594167	0.276502814	0.351494831	-12.9346517
i33.1	-3.0740266	-1.98548566	-6.84707933	-1.54248685	-2.30241617	-11.263106
i33.2	-1.8958909	-1.34484366	-5.35915367	-2.43793785	-0.84984217	-11.1638813
i33.3	-4.0586226	-2.171551	-5.69045967	0.005948814	0.385319831	-7.00872133
i33.4	-2.2700592	-3.51703866	-7.16483633	-0.32007585	-0.2055325	-10.061845
i34.1	-1.2837209	0.661563669	-5.746219	-1.10476819	-1.1092945	-12.5229083
i34.2	-3.3915622	0.317884669	-5.24054433	-0.43970485	0.293305831	-10.877071
i34.3	0.05315942	0.090930669	-4.46477633	1.703077481	1.895154164	-12.3969963
i34.4	0.91110942	2.299931003	-2.680628	4.914099481	3.554076164	-9.72538133
i35.1	3.18223008	1.192799669	-3.605015	0.501222481	-0.68019317	-11.4395813
i35.2	2.87374142	0.508529003	-2.47745967	1.611011147	1.379642164	-9.23464967
i35.3	1.67672508	-2.29961533	-3.86490633	1.420169481	-0.03580184	-6.95814833
i35.4	2.28050375	-0.13578566	-2.295046	2.823993147	2.219958164	-5.96903167
i36.1	0.40754575	1.339097336	-1.534647	0.578497147	1.078142831	-7.311215
i36.2	-0.0188702	-0.085321	-3.71319333	0.434960147	0.512288831	-7.71095567
i36.3	2.46800375	-3.25683133	-4.20464833	-0.01555919	0.045493831	-7.92863267
i36.4	-2.2615242	-2.52324933	-2.680637	0.383220147	-0.41947417	-5.96741967
i37.1	1.75428242	-0.15919866	-4.08549867	1.123719147	-1.39195917	-8.63571133
i37.2	0.46804208	-1.28854166	-3.77217867	0.586475814	-0.20194317	-7.19300867

i37.3	-0.6135109	-1.69149533	-2.802701	1.005376481	-0.67412017	-7.80128967
i37.4	-0.6805562	-0.69061966	-2.77188367	1.789651481	0.587404831	-8.018832
i38.1	2.03978775	-0.25634433	-4.59528167	-1.31610985	-0.03137217	-11.3372547
i38.2	-1.1681296	-2.388214	-3.92117233	-1.51427185	-0.7231415	-8.84704567
i38.3	0.35598442	-2.90279533	-2.60826733	-0.41839852	-0.24996484	-6.791707
i38.4	-2.8153326	-0.85173433	-2.52081367	0.430884814	0.687456497	-8.470796
i39.1	-3.1193799	-0.00583466	-5.64654067	-0.87062519	-0.9104365	-10.57479
i39.2	-3.0617996	-0.65415366	-5.474876	-0.50590485	-0.1978425	-9.39866733
i39.3	-3.7555872	-2.75442533	-5.285979	2.333719814	-0.7155575	-6.98066267
i39.4	-4.1183926	-0.902794	-4.43237267	1.210516814	0.659681831	-8.48097533
i40.1	1.52781908	0.448270336	-4.20017033	-0.25466985	-1.2242805	-10.5594367
i40.2	0.56441675	0.373428003	-2.62811133	1.738487147	0.973257497	-8.861763
i40.3	-0.9274842	-1.28222233	-2.698233	0.249562481	0.786450497	-7.29516933
i40.4	-1.8355884	1.593637669	-1.50108	0.096274147	0.793561497	-9.4461635
i41.1	-0.4904059	3.024311669	-5.12414433	0.788210814	0.715446164	-8.00366633
i41.2	-0.5294296	2.471903669	-5.99613667	-0.78910619	0.500251164	-11.386485
i41.3	-0.0258892	2.537961336	-2.768879	1.049196147	3.100633831	-7.12573933
i41.4	-1.7048386	-0.20904233	-7.371727	-3.51438885	-1.95057984	-15.0141687
i42.1	1.62893975	2.823608669	-5.99849567	0.587099147	-0.1127555	-10.7283223
i42.2	-0.9266132	3.211716003	-5.43137233	0.601337147	0.904124164	-9.16101767
i42.3	-0.1741072	1.380076003	-4.43327667	0.512426147	1.346950164	-10.492439
i42.4	-2.1006436	1.648932003	-4.36166433	1.244849814	0.797225831	-9.51901233
i43.1	2.22695508	-0.49859933	-5.93285533	-0.44876119	-0.5963995	-10.313723
i43.2	-2.1192572	-0.657095	-5.86682633	-1.48754419	-0.6217465	-9.54124533
i43.3	-0.1273439	-1.848134	-3.417252	0.935944814	2.090233164	-4.431944
i43.4	-1.6473269	-2.14518933	-3.59860667	0.516048147	1.405718831	-5.597461
i44.1	0.87219342	0.082091003	-6.784113	-0.34498752	-1.03321017	-9.89634433
i44.2	-1.1082086	1.351795003	-2.19725567	-1.25678385	-1.69969984	-9.842611
i44.3	1.52691808	-0.94863033	-4.18691167	-0.20989685	0.919856831	-11.468624
i44.4	-2.9642799	-0.47549033	-4.35721867	0.494560814	0.763100164	-11.7810087
i45.1	-3.3107279	0.777733003	-7.428115	-1.04109519	-0.5909575	-7.61074667
i45.2	-2.3449906	-3.23982166	-6.31317133	-1.63820635	-0.45146084	-13.9490983
i45.3	0.69185042	-2.00477433	-6.33625267	1.675577481	1.031851497	-13.7410003
i45.4	-2.2240829	-1.47754566	-0.10045633	1.613681814	0.561714831	-11.168154
i46.1	-2.5859949	-2.21005566	-5.859114	0.005737814	2.183474164	-12.054777
i46.2	-1.1326839	-0.95945266	-4.629452	0.914002814	1.152399497	-10.0978605
i46.3	0.78266342	-5.750146	-4.39671167	-2.73578885	1.664510664	-12.1992377
i46.4	-1.3990532	-0.85668766	-3.62166083	-2.82937685	1.903943997	-12.9786693
i47.1	1.42419742	2.834018003	-2.61117633	3.030925814	2.045620497	-9.61466567
i47.2	-1.6155989	1.352034669	-4.09653967	0.746300147	1.545545831	-12.5938413
i47.3	-1.5969632	-1.49665833	-4.35826767	-0.30157852	0.971255497	-6.10196667
i47.4	-3.3001746	-0.57475833	-3.76485533	0.837995481	1.449531164	-7.16407433
i48.1	-3.8834716	0.877339003	-3.53669233	-0.12122385	0.200212831	-11.312721
i48.2	-1.7198846	-0.682172	-4.72771867	-0.63781519	0.518156831	-11.572955
i48.3	-2.1499236	-1.833147	-5.29402733	0.247936481	1.218483497	-10.128129
i48.4	-3.7710739	-2.775848	-6.36829	-1.48980752	-0.0167185	-11.7048947
i49.1	0.47425575	0.502948003	-4.95250533	-0.96539585	-0.26280117	-11.6261487

i49.2	-4.6408506	-1.442035	-5.15598267	-2.78371385	-1.36320617	-12.3766603
i49.3	-3.1738319	-1.11234633	-3.095297	-1.75237052	0.349835497	-8.096598
i49.4	-2.8714952	-2.53751233	-4.58451867	-1.67687419	-0.78808417	-8.490333
i50.1	0.44905408	-0.72581866	-5.03516933	-0.56232519	-1.4435945	-10.4934683
i50.2	2.07119342	-0.54003366	-4.720978	0.155650814	2.083717164	-12.636345
i50.3	-5.3967036	-6.36298233	-9.874873	-2.23297885	-0.84321717	-10.016166
i50.4	-0.7877829	-2.04582533	-4.38778267	0.930964481	3.013911497	-7.782118
i51.1	-2.2458359	-4.31450366	-6.88352967	-3.16361885	-1.14023184	-15.8951457
i51.2	-2.6343772	-3.83467966	-6.86614933	-3.34105752	-1.06470317	-15.177556
i51.3	-4.3344609	-2.954134	-4.171536	-0.81780385	-0.9017625	-14.8120473
i51.4	-0.4426869	-0.44990583	-3.09843467	-1.78852552	1.037418164	-15.0536347
i52.1	-3.4155716	-1.43498266	-5.11591667	-1.46825619	-0.79877317	-13.1140667
i52.2	-2.0225576	-1.478006	-4.371164	-2.22160919	1.217916831	-9.95357033
i52.3	-8.1987612	0.699876003	-0.85065567	2.509601147	2.838050164	-5.865081
i52.4	-1.3432919	-2.00578233	-2.728093	0.173564147	1.730498497	-11.8408667
i53.1	1.84148175	-1.27605133	-5.13277433	0.786343481	3.532108831	-10.7810417
i53.2	-2.8239802	-0.25694733	-5.04868367	0.264453147	0.777267164	-11.0325253
i53.3	11.9883401	3.635919336	-0.115556	4.110690147	4.565061497	-10.1777263
i53.4	-2.6796449	1.140389003	-2.72249533	2.177079147	2.395011164	-10.3279897
i54.1	11.9883401	0.370399003	-5.36534733	0.288899147	1.879280831	-12.722864
i54.2	-4.1660156	-2.59929666	-8.011147	-0.98213919	-3.10016584	-13.8201717
i54.3	-2.4297839	-1.41183533	-5.19965333	-1.44114619	-1.80499984	-10.5527543
i54.4	-4.2389296	-1.26053666	-5.37489233	-0.75092052	1.343459497	-13.180843
i55.1	0.65528575	0.410779669	-3.83508167	0.564139147	3.137862164	-10.7504487
i55.2	-1.4491322	-2.99990633	-5.404284	-1.43825252	-0.0801005	-13.636971
i55.3	-4.1037192	-5.18494633	-9.32612867	-1.48200752	-1.8402255	-19.0017293
i55.4	-1.7828262	-3.65496366	-6.739748	-0.54513619	-0.84371117	-16.6773167
i56.1	-2.9635739	-2.740316	-6.01373733	-1.94777785	-3.16712317	-12.9285433
i56.2	-0.5484112	2.067151336	-2.21364433	1.062165814	1.794689497	-8.202504
i56.3	-1.6580869	-2.550286	-5.507606	-0.17394619	0.405106164	-9.18230567
i56.4	-1.2410649	-0.36573666	-3.48116967	1.516671814	1.760721497	-9.54309833
i57.1	0.54355775	0.819614003	-6.10251367	-0.95207985	-0.76934484	-11.3515027
i57.2	-0.1792662	0.063986669	-5.78490767	-0.58293519	0.782974831	-12.3311173
i57.3	-1.4980662	-2.00751333	-6.10503833	0.925985481	1.238518831	-9.016968
i57.4	-1.4842289	-1.66245733	-4.205635	0.691985481	1.588761831	-9.29672467
i58.1	-3.9637522	-2.54756833	-8.60917333	-4.56728585	-2.04438517	-14.0078813
i58.2	-2.5060809	-0.180705	-7.00034133	-2.37150619	-0.41217117	-12.617135
i58.3	-0.4221756	0.486412336	-3.303514	1.317877814	2.651405497	-12.3560433
i58.4	1.35706975	1.425702003	-2.13703767	1.118712814	2.691111164	-10.298401
i59.1	0.35871108	-1.813489	-5.394347	0.122043147	2.810280497	-11.57185
i59.2	-2.6621459	0.243667669	-6.62246733	-1.32947685	-3.1857165	-13.3371457
i59.3	-4.4553482	-1.26802833	-4.579764	-0.55919052	-0.5282355	-13.2512507
i59.4	-3.3184946	0.259335003	-3.24746167	0.962176814	0.679378164	-11.3316367
i60.1	-0.3213112	-3.23555233	-8.294322	-3.34850985	-0.2084535	-14.5378
i60.2	-0.3888709	-1.46337833	-4.337829	-0.50578485	2.548524831	-11.8185087
i60.3	-1.5012412	-1.10259966	-4.94933433	2.032032814	-0.49232884	-9.11171967
i60.4	0.02742342	2.084061003	-2.61293567	3.192159814	1.366361497	-8.257279

i61.1	2.10807608	-0.06684933	-3.63895867	1.442656481	3.928085164	-10.901059
i61.2	0.08506208	-4.16888483	-5.84266633	-0.50966252	1.415190831	-13.0124963
i61.3	0.39203775	-3.520053	-4.73133033	0.971647814	2.695703831	-11.1713453
i61.4	1.68450175	-2.79724266	-4.59507733	0.800638481	2.303505831	-12.137077
i62.1	-1.2008956	0.925746336	-4.62888633	2.453996481	-1.56724917	-10.924756
i62.2	3.75693742	-1.88999733	-4.772106	2.132600814	4.637576831	-10.700211
i62.3	-2.5153346	0.910634003	-3.72749833	-0.68202552	1.302519164	-9.77529767
i62.4	-3.0335012	-2.20906066	-5.49967667	-1.32139585	-0.26434384	-11.8468207

normalized miR-31-5p	normalized miR-4687-3p	normalized miR-6786-5p	normalized miR-93-5p	normalized miR-1260b
2.7506008	0.767502536	-0.573701908	4.083523369	4.083523369
1.378672133	0.282105869	-0.746336575	2.172623369	2.172623369
3.559204467	1.398822536	-1.153855575	4.043553036	4.043553036
4.834822467	3.810537869	-0.526982575	4.128036703	4.128036703
1.1330758	-0.94861613	-1.880188575	1.342766369	1.342766369
0.349092133	-1.0370218	-1.718116908	1.894810036	1.894810036
1.495570467	0.312442869	0.066987425	3.734310036	3.734310036
3.3720208	0.502623203	-0.716492242	4.824991703	4.824991703
0.5101298	-1.0750208	-0.972286242	0.962755369	0.962755369
0.6104138	-2.13041913	-3.817351575	1.492220036	1.492220036
1.1765368	-0.7439148	-0.321955908	2.425923369	2.425923369
1.3324858	1.105474869	1.417542425	3.696905369	3.696905369
-0.61860487	-0.39353146	-2.176983908	0.974820369	0.974820369
-0.48568587	-1.30766613	-3.225725575	-0.551976631	-0.551976631
3.1928498	0.805152869	-0.134951575	3.370427036	3.370427036
-3.9496932	-0.18034013	-2.834754908	1.211826703	1.211826703
4.711542133	7.563197536	3.586659425	5.585508703	5.585508703
2.7245648	6.755982536	1.589482425	3.192896703	3.192896703
0.159037467	0.965538536	-2.059284242	3.298583703	3.298583703
0.848541467	2.548358869	-0.138547575	2.535402703	2.535402703
1.257502467	1.214653536	-2.622081908	2.606427036	2.606427036
0.9155288	0.714540536	-2.082270908	1.507119036	1.507119036
2.9995858	3.402930869	-0.144631575	2.437290369	2.437290369
2.7728658	2.992306536	-1.024679575	2.006343703	2.006343703
3.4179768	8.675012869	3.741860425	3.731446036	3.731446036
1.873601467	5.202755536	1.679555092	2.018107703	2.018107703
-3.0906072	2.390493869	-0.208290242	1.360025036	1.360025036
-3.76856487	4.114347869	0.873049425	1.301937036	1.301937036
2.7954378	7.451839869	4.553062758	3.508215036	3.508215036
-0.13969087	1.701484869	-2.292567242	0.397866703	0.397866703
3.436264467	4.128054869	0.888718092	2.423776703	2.423776703
1.2194488	3.132671536	-1.042936575	0.853565369	0.853565369
-0.79486853	3.948291869	1.569748092	3.134118036	3.134118036
0.165364133	2.489381869	1.164783092	1.508213036	1.508213036
-0.3109202	3.311310536	0.050975092	2.371581036	2.371581036
1.822197133	6.674273536	4.366884092	4.076239369	4.076239369
-0.71182753	2.239016203	-1.186767575	4.359453369	4.359453369
0.7665668	2.572519203	-1.151577242	3.573964036	3.573964036
-0.61736187	2.436041203	-1.357098575	3.087431369	3.087431369
0.3697818	1.488593869	-1.310232575	3.218047703	3.218047703
0.290087133	4.931750869	1.813676092	1.938540703	1.938540703
-0.82987053	1.867963203	-1.561192242	0.927344703	0.927344703
3.551667467	3.600767869	0.411188758	3.106140036	3.106140036
2.222530467	2.231628203	-1.013175908	1.435305703	1.435305703

1.3177518	2.767092203	-0.060865908	4.107059369	4.107059369
1.6355748	1.679205536	-0.709167908	3.933029036	3.933029036
2.2143538	2.361577869	-0.985877908	3.356761369	3.356761369
3.204085467	5.724376869	1.899065425	3.614535369	3.614535369
0.553090467	3.810276536	2.760281092	1.476910369	1.476910369
-2.58191487	-0.0882818	-1.136698242	-0.033715631	-0.033715631
2.600415467	3.002735203	1.678021425	2.181575036	2.181575036
2.265711467	0.214941203	-1.522018575	1.201109703	1.201109703
-1.7177012	-3.38688946	-2.659580908	-1.335561631	-1.335561631
0.370721467	-3.7833658	-2.729707575	-1.478733964	-1.478733964
4.745123133	0.148078869	4.779330092	3.478047036	3.478047036
3.0498798	1.172347869	4.871630092	3.261676036	3.261676036
-2.75228503	-6.3314643	-4.211831075	-4.999181797	-4.999181797
-1.86906812	-3.85023638	-3.127390825	-2.891778214	-2.891778214
0.171479467	-1.97047363	0.077719425	0.427194036	0.427194036
-2.63396141	-4.14660567	-3.358392783	-2.255332172	-2.255332172
-1.01961953	-2.89384646	0.651485092	0.003681369	0.003681369
-1.18895087	-3.04919346	-2.004570242	-1.951159631	-1.951159631
-5.50041087	-1.38131813	-0.971676242	3.204316369	3.204316369
-5.58843487	-2.87033946	-1.091780908	1.363823369	1.363823369
-0.5249132	-3.8689638	-0.757723575	-0.710000631	-0.710000631
0.970730467	-2.91124546	-1.817102242	-0.900804631	-0.900804631
2.509751467	0.152954869	1.903984425	1.738900036	1.738900036
2.6993248	-0.72013146	2.497572425	1.992922036	1.992922036
0.8295568	-3.08973413	-0.017818908	-1.270928297	-1.270928297
-0.92491587	-2.90144713	-0.080972575	0.684093703	0.684093703
-2.9274122	-1.62695246	0.782829092	2.386401369	2.386401369
-3.47026487	-1.0067558	0.263524092	0.766822369	0.766822369
0.2458008	-2.2613288	-0.072814242	0.451706036	0.451706036
-0.49048387	-2.72027113	-1.695314575	-1.043988631	-1.043988631
-4.12984187	-0.98566246	1.721658425	1.846519369	1.846519369
-4.6868512	0.971426869	2.361925758	2.640818703	2.640818703
-1.47270487	-1.87231413	0.287643425	-0.193187631	-0.193187631
-1.16363453	-2.5390608	0.045876425	-0.922605631	-0.922605631
-1.3380782	-2.79532713	0.051164758	1.161009369	1.161009369
-3.86593653	-3.4892288	-0.882494908	0.123511036	0.123511036
-1.3699332	-3.24549246	-1.820694908	-1.437978297	-1.437978297
1.063578133	-0.5835838	1.165794425	0.569438036	0.569438036
-0.89197253	-1.8795098	-0.292917242	-1.709521964	-1.709521964
2.2888008	-1.2151498	-0.148341242	-0.004851964	-0.004851964
-0.16289887	-3.4368968	-1.846078242	-2.352341297	-2.352341297
1.997438467	0.224623869	1.116447092	0.064904036	0.064904036
0.1062718	0.258141536	0.674771425	-1.919536631	-1.919536631
3.612995133	2.434381203	1.864501758	1.299903703	1.299903703
-0.1936122	1.017521203	-1.362684575	-0.634721631	-0.634721631
-2.17172153	1.170222536	1.420231758	0.140664036	0.140664036
-6.0261632	-1.35331613	-1.402821908	1.998116369	1.998116369

-3.64689153	2.843328203	0.620779092	2.605625703	2.605625703
-1.40754987	-1.7971778	-2.267436242	-0.533190964	-0.533190964
-0.95077787	-2.73010813	-3.664583742	-2.891505297	-2.891505297
-1.29776953	3.363901536	4.531192092	2.238645036	2.238645036
-1.6450252	-0.69128546	-1.190402242	0.388351369	0.388351369
-0.70905087	-2.26205146	-2.952225242	-1.649610964	-1.649610964
-2.56161587	-3.40357146	-3.989530908	-2.800944964	-2.800944964
-0.33620787	-3.0851298	-1.248057242	1.233501703	1.233501703
-2.4664802	-2.31272246	-2.803955575	-0.828386297	-0.828386297
0.474664133	0.058736203	2.597109092	0.241537703	0.241537703
-0.92336287	-0.40627446	-0.688332575	-0.418135631	-0.418135631
-4.5754282	0.071236203	-0.330996908	1.780890703	1.780890703
-6.56096687	1.246898536	-0.721844575	2.568589036	2.568589036
0.265135133	-2.03647513	-0.578754908	-2.094785297	-2.094785297
1.317100467	-1.46632313	0.322194425	-2.422006631	-2.422006631
3.455430467	-0.94070613	0.221333092	-1.791488631	-1.791488631
4.924823467	0.561825536	2.797416092	-0.887941964	-0.887941964
-0.71596587	-1.74867646	-1.966027575	-3.812567964	-3.812567964
0.9056638	-1.72393446	-2.256783908	-2.833010964	-2.833010964
3.51987547	-2.36305946	-1.369234572	0.460780036	0.460780036
2.8655858	-0.3257088	1.134327092	1.605587036	1.605587036
-1.65058053	-1.1045898	-0.450054575	-2.740471631	-2.740471631
-0.16455487	0.680588203	-0.822498242	-1.414127297	-1.414127297
1.4704278	1.128237536	0.031993425	0.171851703	0.171851703
3.5028788	2.001842869	0.460955425	-1.142920964	-1.142920964
2.369402133	1.670392869	0.581166758	-0.715336631	-0.715336631
2.687818133	-0.79377913	-0.646520575	-1.615205631	-1.615205631
-2.2574492	-0.1519038	-0.630640575	-0.034886631	-0.034886631
-3.51106453	1.155436203	-1.044018242	-0.042077631	-0.042077631
-0.70637203	-2.42797946	-2.372248242	-2.248704964	-2.248704964
0.006728133	-2.14693713	-1.297167575	-0.699885631	-0.699885631
2.270314133	-2.51634646	-3.154291575	-0.608040631	-0.608040631
0.062307467	1.841887536	-2.527264242	-0.022180964	-0.022180964
-0.75143587	-1.86649513	-2.485319908	-0.974640631	-0.974640631
0.638633133	-1.7766708	-1.651612575	0.040727703	0.040727703
-2.5150392	2.918222536	-0.126927242	1.365438703	1.365438703
2.205925467	5.813585536	1.885852092	3.592866703	3.592866703
0.662959467	0.576734203	0.658733092	-0.590122631	-0.590122631
4.016840467	-0.6135198	1.266455758	0.160025369	0.160025369
1.106154133	-0.4266538	0.627797425	0.930945036	0.930945036
3.8700268	1.183469203	2.247900425	2.387251369	2.387251369
4.227133467	1.010154536	1.848402092	-0.666565964	-0.666565964
3.043052133	-0.63050313	-1.344909575	-1.476350297	-1.476350297
2.407606467	1.310945869	0.427452758	-0.509785964	-0.509785964
2.578356133	-1.09077613	-1.745212575	-1.496172964	-1.496172964
0.288688467	-0.89031113	0.301023425	-0.679731297	-0.679731297
1.291253133	-0.62440246	-1.886475242	-1.173224631	-1.173224631

0.2340048	-1.7120138	0.145597425	-0.457380297	-0.457380297
-0.36548053	-0.58981213	-0.024584908	-1.395391631	-1.395391631
0.442704133	-0.40813846	-1.298266575	-1.846091631	-1.846091631
-0.62093853	-2.10672513	-2.912570908	-2.323372964	-2.323372964
1.008159467	-1.62323613	-2.546368908	-1.165784297	-1.165784297
-0.13596887	-0.9058758	-2.010848908	-2.058217964	-2.058217964
0.457798133	-2.8623238	-1.747044242	-0.474336297	-0.474336297
0.8135978	-1.97194946	-0.807779242	-0.214103964	-0.214103964
2.1953898	-1.39074013	-0.958859908	1.789008703	1.789008703
2.0926798	-1.49257546	0.022712092	1.147806369	1.147806369
0.504812467	0.411411869	-0.332022575	-1.755537631	-1.755537631
2.187351467	1.845330536	1.706508425	-0.748718297	-0.748718297
2.2083518	-1.7593328	-0.037324908	-0.191055631	-0.191055631
0.6107208	-0.0869408	0.851916092	-1.347133964	-1.347133964
1.196276467	-2.64710513	-0.149507575	0.994134369	0.994134369
1.411170467	0.675198536	1.073408758	0.108951036	0.108951036
3.519936467	1.240384869	1.200517425	1.567114703	1.567114703
-2.08007053	-3.76613913	-3.877296242	-2.808876964	-2.808876964
-0.65123287	-1.14830913	0.233173092	0.883834036	0.883834036
2.792534133	-0.2330718	1.717705092	0.463530369	0.463530369
-0.42485487	-0.08397513	1.580900092	0.288245703	0.288245703
0.5475168	-0.2660158	0.138766425	0.389523369	0.389523369
1.461954133	-2.2479968	-1.036562908	0.769654036	0.769654036
0.774026133	-1.85942646	-2.097905908	-0.714098964	-0.714098964
5.470825133	-0.22297846	0.228086092	1.759012036	1.759012036
3.349280133	0.206583536	1.220110758	1.112006036	1.112006036
1.089419467	-1.90823846	-1.723195908	-0.310767297	-0.310767297
0.362018133	-2.0372398	-4.453884908	1.707584369	1.707584369
-1.34340153	0.195895869	-0.638846908	0.288040703	0.288040703
-1.55117987	-2.15876413	-1.667465908	0.260734369	0.260734369
1.207669133	-2.20360313	-1.684322242	-0.217447631	-0.217447631
-0.97989553	-0.67348213	-0.647972908	0.510413369	0.510413369
-5.56385387	-1.54700846	1.781213092	0.025319703	0.025319703
-2.4556922	-2.20132713	0.095703092	0.022809369	0.022809369
-1.7164322	2.978402536	3.623939425	0.316553369	0.316553369
0.9794288	-0.91993713	1.987631758	-0.394361964	-0.394361964
-3.47814053	0.382352203	-6.935726575	0.585694536	0.585694536
-3.1445212	0.299161536	-2.024632908	-3.849911631	-3.849911631
2.965639467	2.111888203	2.696212425	1.641522703	1.641522703
2.618039467	-0.58973163	0.344718092	0.463155203	0.463155203
4.478345133	0.637516203	-0.778029908	0.308150036	0.308150036
4.527606133	0.673284869	0.179751092	0.891494703	0.891494703
-0.5109492	0.319769536	2.350792425	-0.696634631	-0.696634631
-0.02411053	0.583449203	0.295335425	-1.468055631	-1.468055631
1.4706708	-1.39948446	-0.398058575	0.578094703	0.578094703
-0.3861282	-2.16837913	-1.136470242	-1.948885297	-1.948885297
-0.0969052	-0.90604113	-0.149117908	-0.634821297	-0.634821297

-1.84733187	-1.41546946	-1.794020242	-2.970852297	-2.970852297
0.7471238	-1.30364313	-1.617543408	-0.653236297	-0.653236297
0.973853133	-2.20073813	-3.632141242	-1.241278297	-1.241278297
-2.13184653	-1.85571146	0.666662425	-1.081953297	-1.081953297
0.9770718	2.746827203	3.520753092	1.559173036	1.559173036
-0.2671912	-4.1329138	-1.659436908	-2.432821297	-2.432821297
3.180800467	-1.60524746	-1.722159575	1.253340036	1.253340036
-3.31783387	0.240211536	0.659294425	-0.843607964	-0.843607964
-1.68148787	0.369008536	0.826798092	-1.811820297	-1.811820297
-3.24342737	-2.81358546	-4.057868908	-2.164431631	-2.164431631
-3.14624653	-2.9274598	0.119416092	-0.914590297	-0.914590297
-1.83267353	-2.27830813	-3.293451575	-1.121458964	-1.121458964
-0.05772453	0.471136536	-0.510422242	-1.461732631	-1.461732631
3.9294468	3.169167869	1.569825758	1.494070036	1.494070036
1.787754133	3.488050203	2.177711092	1.874658369	1.874658369
1.341581467	2.618223869	4.942919425	1.680669036	1.680669036
0.3899788	1.163906203	-1.184168242	0.730683036	0.730683036
2.297959467	6.239515536	5.286959425	3.293382369	3.293382369
0.340798133	2.206130536	1.433515425	2.224531703	2.224531703
-3.07403087	3.325182536	2.991463758	0.093063036	0.093063036
-1.95036453	-2.6212838	-5.339294908	-3.286628297	-3.286628297
-0.60439053	-1.3665198	-4.693887908	-0.559161297	-0.559161297
0.138912133	1.034889536	2.810386092	0.560760703	0.560760703
1.332942133	2.614258536	4.087722758	2.236534036	2.236534036
-0.83822187	0.607867203	2.139291425	0.022585703	0.022585703
-6.87294687	-1.9789268	-4.924413242	-1.014640297	-1.014640297
-5.64782887	0.882535536	-3.298014242	-2.248426297	-2.248426297
-1.87982553	-3.67001913	-4.404263242	-2.420353631	-2.420353631
1.877064133	2.445574869	1.443358758	-0.397525631	-0.397525631
0.206648133	-1.4816188	-0.621079242	-0.469330631	-0.469330631
0.3901428	0.296444203	1.667947092	-0.780014297	-0.780014297
-1.48345187	-1.46536013	0.544634092	-1.259665297	-1.259665297
-1.63209153	1.273408536	0.427885758	-0.905643964	-0.905643964
0.597051467	1.271860869	-1.170999242	0.252395369	0.252395369
0.594373133	2.247389869	0.708243425	0.984723703	0.984723703
-3.60082053	-1.89409546	-4.423321242	-3.357491964	-3.357491964
-2.97935987	-0.01989846	-0.955724242	-1.990596297	-1.990596297
-3.0157972	0.818940536	-0.092846908	2.400319036	2.400319036
-0.96485653	-1.30310113	1.052320758	0.674332369	0.674332369
0.925875133	2.712201203	2.955537425	0.563081036	0.563081036
-1.83203753	-0.7668388	-3.065358242	-1.759115631	-1.759115631
-2.59377753	0.954148869	-2.657561908	0.459918036	0.459918036
-0.59795753	2.279778536	-1.182486242	1.731084369	1.731084369
-5.00584553	1.904020203	1.576794092	-3.206680631	-3.206680631
-1.44027053	2.577633203	3.648425092	-0.043418297	-0.043418297
0.810363467	-0.2865118	-4.440286242	0.653101703	0.653101703
2.9211028	2.365104869	-1.002846908	2.320889036	2.320889036

0.572744133	4.368903869	5.065093425	1.115746036	1.115746036
-0.94550687	1.934021536	2.351808092	-0.456866964	-0.456866964
0.254134133	2.403032536	3.935836758	0.591288036	0.591288036
1.256771133	2.804991869	4.030393092	0.552070036	0.552070036
0.981820133	1.126458536	-1.809844908	0.063217369	0.063217369
0.709035467	3.779521203	5.434984425	0.817103036	0.817103036
-0.6789522	1.453803869	-1.432468908	-0.059702631	-0.059702631
-1.77119453	-0.5861838	-4.824477908	-1.588585964	-1.588585964
