# Supplementary material for: Global microRNA expression profile in laryngeal carcinoma unveils new prognostic biomarkers and novel insights into field cancerization
Source: Sci Rep. 2022 Oct 12;12:17051. doi: 10.1038/s41598-022-20338-w (PMC9556831; doi:10.1038/s41598-022-20338-w)
Supplement: Supplementary file 5 — Supplementary Information 5. [file 41598_2022_20338_MOESM5_ESM.docx]

**Supplement 1**. MicroRNAs with significantly dysregulated peritumor laryngeal mucosa consistent with expression dysregulation in tumor samples. The analysis is based on microarray data.

**Supplement 2**. MiR-1260b, miR-21-3p, miR-3,1-3p and miR-31-5p exhibited significantly different expression levels in the tumor grade comparison.

**Supplement 3**. Venn diagram of the microRNA distribution of our dysregulated microRNAs overlapping among different microarray profiles published in the literature.

**Supplement 4.** Raw data table.
